# Carp Edema Virus Infection Is Associated With Severe Metabolic Disturbance in Fish

**DOI:** 10.3389/fvets.2021.679970

**Published:** 2021-05-19

**Authors:** Jiri Pikula, Lubomir Pojezdal, Ivana Papezikova, Hana Minarova, Ivana Mikulikova, Hana Bandouchova, Jana Blahova, Małgorzata Bednarska, Jan Mares, Miroslava Palikova

**Affiliations:** ^1^Department of Ecology and Diseases of Zoo Animals, Game, Fish and Bees, Faculty of Veterinary Hygiene and Ecology, University of Veterinary Sciences Brno, Brno, Czechia; ^2^Department of Zoology, Fisheries, Hydrobiology and Apiculture, Mendel University in Brno, Brno, Czechia; ^3^Department of Infectious Diseases and Preventive Medicine, Veterinary Research Institute, Brno, Czechia; ^4^Department of Animal Protection and Welfare and Veterinary Public Health, Faculty of Veterinary Hygiene and Ecology, University of Veterinary Sciences Brno, Brno, Czechia; ^5^Department of Epizootiology and Clinic of Bird and Exotic Animals, Faculty of Veterinary Medicine, Wrocław University of Environmental and Life Sciences, Wrocław, Poland

**Keywords:** emerging viral diseases, fish, pathophysiology, electrolyte and acid-base imbalance, hypotonic dehydration, endogenous hyperammonaemia

## Abstract

Significant mortalities associated with emerging viral diseases are challenging the economy of common carp aquaculture. As such, there is an increased need to disentangle how infected fish cope with progressive disease pathology and lose the ability for homeostatic maintenance of key physiological parameters. A natural carp edema virus (CEV) infection outbreak at a carp fish farm provided an opportunity to examine diseased and healthy carp in the same storage pond, thereby contributing to our better understanding of CEV disease pathophysiology. The disease status of fish was determined using PCR-based virus identification combined with analysis of gill pathology. Compared with healthy control carp, the blood chemistry profile of CEV-infected fish revealed major disruptions in electrolyte and acid-base balance (i.e., hyponatraemia, hypochloraemia, hyperphosphatemia, elevated pH, base excess, and anion gap and decreased partial dissolved carbon dioxide). In addition, we recorded hyperproteinaemia, hyperalbuminaemia, hypotonic dehydration, endogenous hyperammonaemia, and decreased lactate along with increased creatinine, alkaline phosphatase, alanine aminotransferase, and aspartate aminotransferase. Red blood cell associated hematology variables were also elevated. The multivariate pattern of responses for blood chemistry variables (driven by sodium, pH, partial dissolved carbon dioxide, ammonia, and albumin in the principal component analysis) clearly discriminated between CEV-infected and control carp. To conclude, we show that CEV infection in carp exerts complex adverse effects and results in severe metabolic disturbance due to the impaired gill respiratory and excretory functioning.

## Introduction

Mass mortalities associated with viral diseases challenge the economy of common carp (*Cyprinus carpio*) aquaculture (1–3). Over the last decade, carp edema virus (CEV) infection, also termed koi sleepy disease, has emerged as a serious disease threatening the European common carp aquaculture and koi trade (1, 4, 5). CEV infections, both in common and koi carps, are currently known from many European countries (1, 4–9). Outbreaks of CEV in the Czech Republic date back to 2013. Since then, CEV genogroups I and II have been detected in this Central European country based on sequencing the 357-bp nucleotide encoding the P4a protein (10). Interestingly, common carps purchased in the Czech Republic for restocking a pond played probably a role in the CEV outbreak in Austria in 2014 (5). While rapid and accurate diagnostic assays are imperative for effective surveillance and control of such infectious diseases (11), advances in recognition of the pathophysiological mechanisms underlying clinical infection signs and mortality improve our understanding of disease progression.

CEV is characterized by lethargic behavior, fish lying on the bottom of ponds, skin lesions around the mouth and at the base of fins, inflammation of the anus, enophthalmos, and swollen and necrotic gills that induce hypoxia and respiratory distress. Mortality may be as high as 100% and adverse effects are believed to increase with the extent of gill pathology. As CEV requires permissive temperatures of between 15 and 25°C, outbreaks tend to occur seasonally in spring and autumn, often affecting new fish following the stress of transportation and restocking (1, 4, 5). Outbreaks of infection may, however, occur also at lower water temperatures (6–10°C), resulting in a protracted course of the disease (9, 12).

Little is known about the blood profile responses of CEV-infected carp as, up to now, only single specimens have been blood-sampled for examination (5) and/or only a few blood profile parameters have been reported (13). Moreover, comparison of blood profile parameters with normal ranges for the species (14) will be of limited value as such parameters are strongly influenced by variations in environmental physical and chemical properties. Finally, where CEV-induced mortality approaches 100%, it can be difficult to obtain comparative data from healthy control fish (i.e., CEV-negative) sampled under the same conditions at the same site.

Recently, we had an unparalleled opportunity to examine CEV-infected and healthy carp in the same fish farm storage pond under conditions of a natural CEV infection outbreak. To better understand CEV disease pathophysiology, we report on blood profile parameters sampled during this outbreak event. It seems reasonable to hypothesize that manifestations of CEV infection in carp, including extreme lethargy, decreased locomotor activity, and enophthalmos, can best be explained by the pathophysiological consequences of impaired respiratory and excretory functioning in the gills, leading to osmotic, ionic, and acid-base imbalance. Alongside extensive necrotic branchial lesions in CEV-infected carp, we predict (1) insufficient oxygen delivery for tissue metabolism, (2) sedative/narcotic effects of metabolic carbon dioxide elevation and respiratory acidosis, (3) neurological abnormalities associated with loss of electrolytes, and (4) dehydration. An improved understanding of how CEV infection alters the homeostatic maintenance of key physiological parameters will have positive ramifications for fish medicine as regards other infections causing gill pathology, such as the koi herpes virus (CyHV-3).

## Materials and Methods

### Outbreak Description

In mid-December 2020, we investigated a case of high mortality in market-size common carp (2,200–2,500 g) kept at a storage pond at the lowland Napajedla fish farm (Czech Republic). Based on available records, it became clear that the first clinical signs of CEV were recognized about 1 month after translocation of about 1,500 kg of fish from a highland pond. No disease had been reported from either site at the time of fish movement. According to the respective fish farm managers, while water temperatures ranged from 10 to 12°C at the highland pond on the date of translocation (16 October 2020), temperatures at the lowland storage pond were around 17°C. Interestingly, the fish farmers also stressed that only translocated fish were showing signs of the disease, including extreme lethargy, shoaling close to the storage pond water inflow, enophthalmos, and gill necrotic lesions. At that point, the same storage pond contained specimens of clinically diseased carp and those showing no clinical signs of the disease. Suspecting a viral infection, we captured 22 carp and took blood samples, following which they were euthanised and subjected to dissection and virological examination. Aquatic environmental quality parameters were measured *in situ* at the time of carp capture (15 December 2020) using an HQ40D portable multimeter (Hach, Loveland, Colorado, United States). The results obtained indicated a water temperature of 4.2°C, oxygen content at 9.46 mg/L and a pH of 7.9. Considering the outcome of CEV infection at the respective farm, while diseased carps were sampled in mid-December 2020, farmers reported 100% mortality of translocated fish by the end of January 2021.

### Virus Identification, PCR, and Sequence Analysis

Gill samples were processed for virus identification using standardized polymerase chain reaction (PCR) methods. DNA was extracted from the gill tissue using the QIAamp DNA Mini kit (Qiagen, Germany), as described in the manufacturer's instructions. DNA extracts were tested (i) for presence of koi herpes virus (CyHV-3), using nested PCR according to Bercovier et al. (15), and (ii) for presence and viral load of CEV (measured as copies of the P4a gene per 250 ng of extracted CEV DNA) using nested PCR and real-time PCR, respectively, as described by Matras et al. (7). Primers used in the present study were as follows: (1) CEV conventional PCR: CEV-ForB: 5′-ATG GAG TAT CCA AAG TAC TTA G-3′; CEV-RevJ: 5′-CTC TTC ACT ATT GTG ACT TTG-3′; (2) CEV nested PCR: CEV-ForB Internal: 5′-GTT ATC AAT GAA ATT TGT GTA TTG-3′; CEV-RevJ Internal: 5′-TAG CAA AGT ACT ACC TCA TCC-3′; (3) CEV qPCR: CEV-qFor1: 5′-AGTTTTGTAKATTGTAGCATTTCC-3′; CEV-qRev1: 5′-GATTCCTCAAGGAGTTDCAGTAAA-3′; Cev-qProbe1: 5′-FAM-AGAGTTTGTTTCTTGCCATACAAACT-3′; (4) KHV qPCR: KHV-86f: 5′-GACGCCGGAGACCTTGTG-3′; KHV-163r: 5′-CGGGTTCTTATTTTTGTCCTTGTT-3′; KHV-109p: 5′-FAM-CTTCCTCTGCTCGGCGAGCACG-3′. PCR products were visualized using the Fast Gene® GelPic Imaging System (GmbH, Germany) following electrophoresis in 2% agarose gel stained by 1× Gel Red (Biotium, USA). PCR products were sequenced by Sanger direct sequencing at a commercial company (SEQme, Czech Republic). The CEV sequences obtained were compared with other 357-bp nucleotide sequences encoding the P4a protein found in GenBank analyzed in a maximum-likelihood phylogenic tree using the Jukes–Cantor model in MEGA v. 6 Software (16), with the robustness of the tree being tested using 1,000 bootstrap replicates.

### Blood Sampling and Measurement

The *caudalis* vessels of 22 carp were punctured with an 18G needle and blood drawn into a 5 mL heparinised polypropylene syringe. Immediately after collection, an i-STAT portable clinical analyser for veterinary use (EC8^+^ diagnostic cartridge based on electrochemical sensing technologies; Abaxis, USA) was used to measure the following blood profile parameters: sodium (Na, mmol/L), potassium (K, mmol/L), chloride (Cl, mmol/L), total dissolved carbon dioxide (tCO_2_, mmol/L), blood urea nitrogen (mmol/L), glucose (mmol/L), pH, partial dissolved carbon dioxide (pCO_2_, kPa), bicarbonate (HCO_3_, mmol/L), base excess (BE, mmol/L), and anion gap (AnGap, mmol/L). The remaining portion of each blood sample was transported to the laboratories of the University of Veterinary Sciences Brno (Czech Republic), where it was used to determine red blood cell count (T/L), hemoglobin (g/L), and hematocrit (L/L), to calculate hematological indices mean corpuscular volume (fL), mean corpuscular hemoglobin (pg) and mean corpuscular hemoglobin concentration (L/L), and, following centrifugation to obtain plasma, Ca (mmol/L), P (mmol/L), Mg (mmol/L), creatinine (μmol/L), ammonia (μmol/L), triglycerides (mmol/L), lactate (mmol/L), albumin (g/L), total protein (g/L), alkaline phosphatase (μkat/L), alanine aminotransferase (μkat/L), aspartate aminotransferase (μkat/L), and lactate dehydrogenase (μkat/L), spectrophotometrically using a Konelab 20i biochemical analyser and commercial test kits (Biovendor, Czech Republic) as described elsewhere (17).

### Data Analysis

Using findings of virology and pathology to assign data to both groups, hematology and blood chemistry parameters measured in CEV-infected and healthy control carp were compared using the Kolmogorov–Smirnov and Shapiro–Wilks tests, one-way analysis of variance (ANOVA) and the non-parametric Kruskal Wallis, Tukey's multiple comparison and Mann–Whitney *U* tests. Levels of significance were set at either *p* < 0.05 or *p* < 0.01. Multivariate analysis of blood profile parameters was performed using principal components analysis (PCA) to differentiate between CEV-infected fish and healthy control specimens. Original measured blood profile data used in this study are available as Supplementary Table 1. All analyses were undertaken using Statistica for Windows® 10 (StatSoft, Inc., USA).

## Results

**Figure 1:**
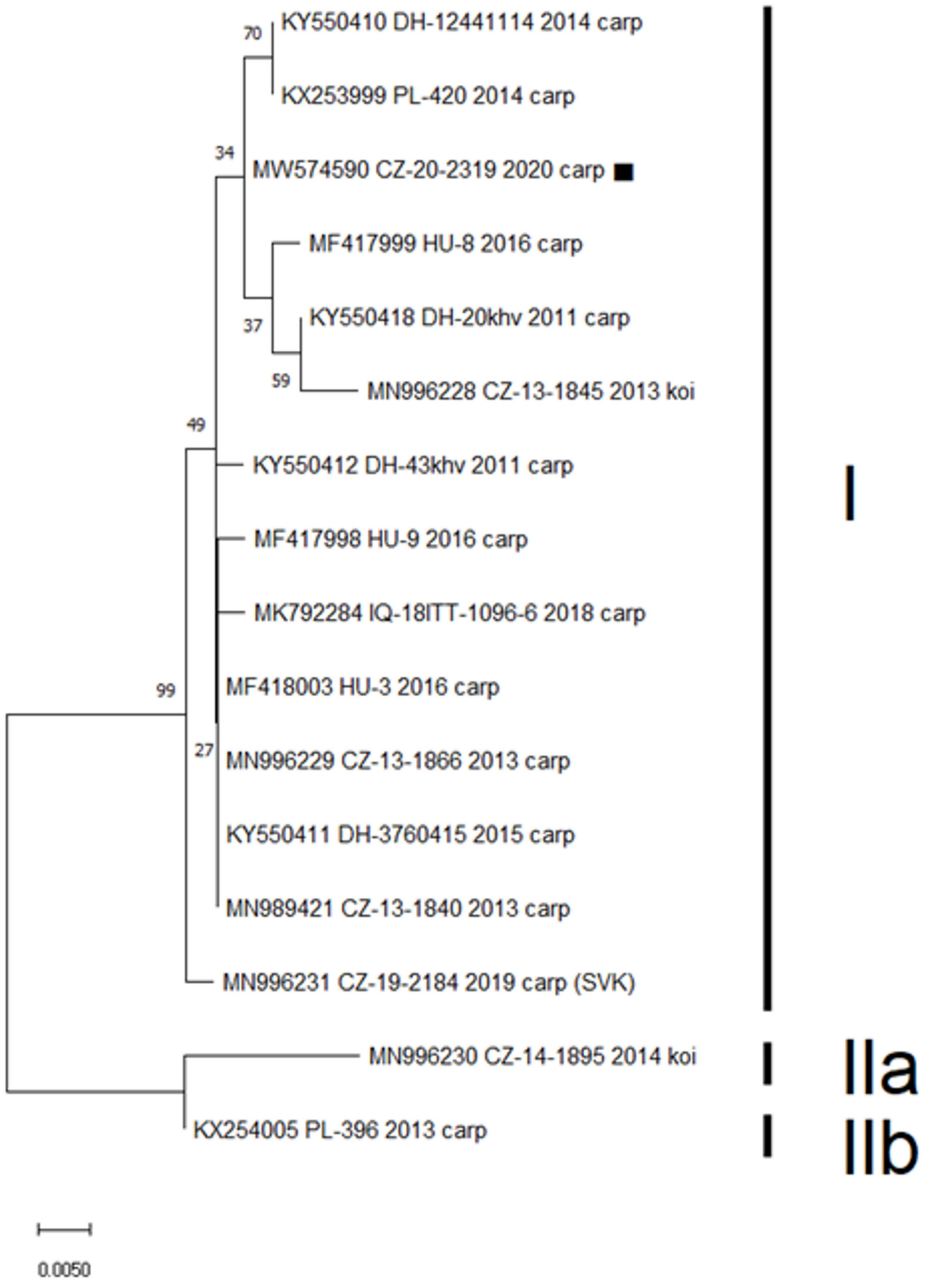
Phylogenetic analysis of a 357-bp nucleotide sequence encoding the CEV P4a core protein. Included was the sequence obtained in this study (MW574590, marked), previously published Czech (CZ) and Slovak (SVK) isolates, and CEV sequences obtained from GenBank with highest identity to the presented case from Germany (DH), Hungary (HU), Poland (PL), and Iraq (IQ). Three proposed CEV genogroups are marked as I, IIa, and IIb. The Maximum Likelihood Tree was constructed using a Jukes-Cantor model and the robustness of the tree was tested using 1,000 bootstrap replicates. The branch length is proportional to the number of substitutions per site.

In total, 13 of the 22 gill samples (59%) proved positive for CEV. Viral loads ranged from 442,228 to 3,036,744 copies of viral DNA per 250 ng of extracted DNA. Sanger sequencing of the product from nested PCR resulted in a 433-bp nucleotide sequence identical for all 13 positive samples. This sequence was uploaded to the GenBank database under the accession number MW574590. Analysis of a 357-bp segment of the sequence assigned the isolate to CEV genogroup I, with five published sequences (originating from Czechia, Germany, and Poland) showing only a single nucleotide difference (Figure 1). None of the samples proved positive for koi herpes virus.

**Figure 2:**
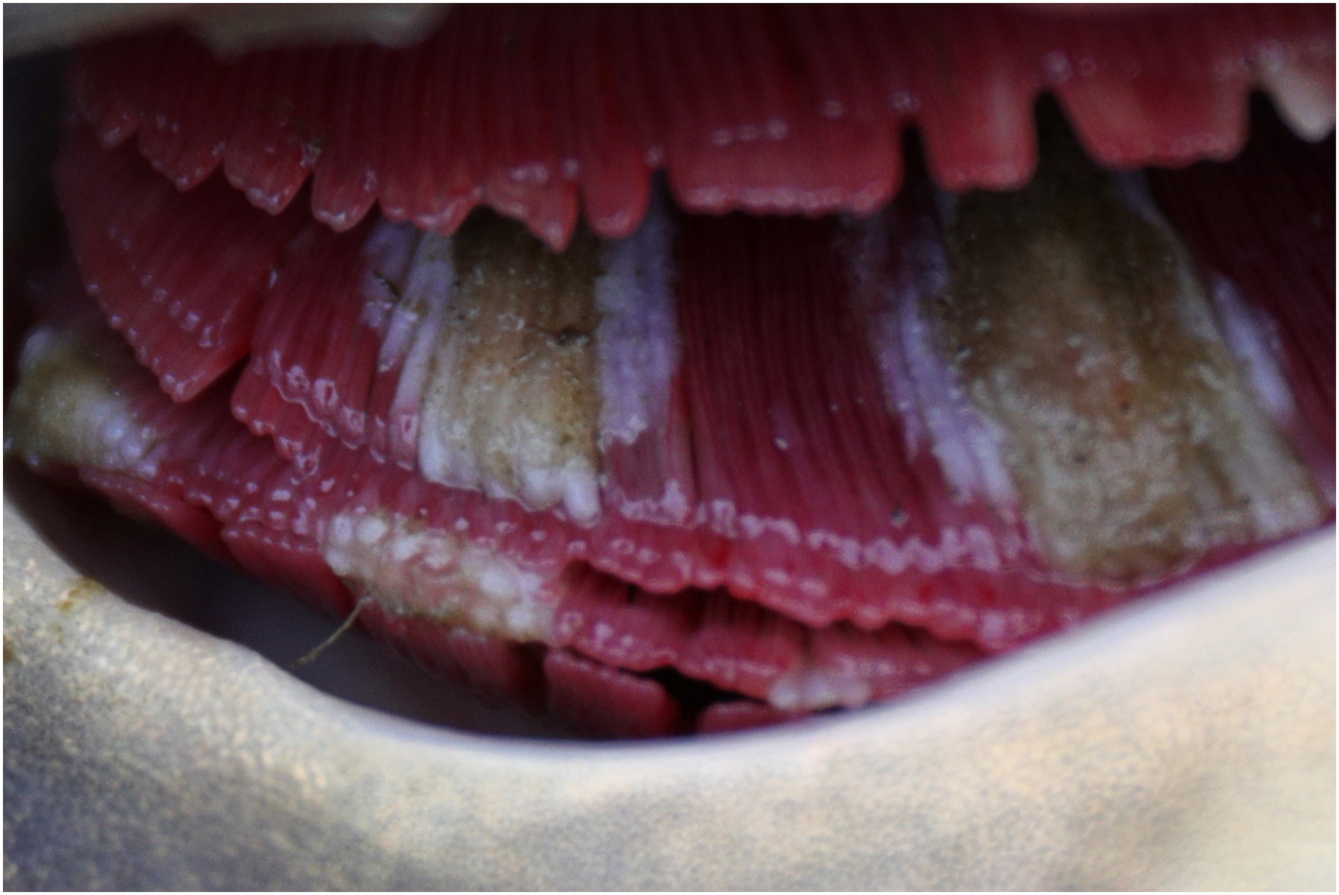
Typical gill pathology observed in carp assigned to the CEV-infected group.

Based on the above virological findings, along with presence of branchial lesions ranging from excessive mucus and gill tissue swelling to extensive necroses with secondary fungal overgrowth (Figure 2), enophthalmos, skin erosions, and/or increased mucus production on body surface on gross examination, blood profile parameters for individual carp were assigned to either CEV-infected or the uninfected control group to allow comparative analysis. Of the 30 blood profile variables measured, 19 showed clear differences between CEV-infected and healthy carp (Table 1). Likewise, there were clear differences in red blood cell associated hematology variables between the two groups. Both sodium and chloride electrolyte values were drastically reduced in CEV-infected carp, while phosphorus levels had increased. Concerning acid-base balance variables, pH, partial dissolved carbon dioxide, base excess and anion gap were all significantly altered in CEV-infected carp, while both protein catabolism products ammonia and creatinine were elevated in CEV-infected fish. Moreover, CEV infection was associated with elevated levels of total protein, albumin, alkaline phosphatase, alanine aminotransferase, and aspartate aminotransferase, and a decrease in lactate.

**Table 1:** Haematology and blood chemistry values measured in carp edema virus (CEV)-infected and control fish (*Cyprinus carpio*).

	**CEV-infected fish**	**Control fish**
**Variable**	***N* = 13**	***N* = 9**
Haematocrit (L/L)	0.36 ± 0.09^**^	0.24 ± 0.05
Hemoglobin (g/L)	94.64 ± 7.93^**^	72.62 ± 15.56
Red blood cell count (T/L)	1.67 ± 0.49^*^	1.13 ± 0.31
Mean corpuscular volume (fL)	208.39 ± 23.89	209.95 ± 31.35
Mean corpuscular hemoglobin (pg)	56.59 ± 5.81	64.03 ± 15.25
Mean corpuscular hemoglobin concentration (L/L)	0.27 ± 0.003^**^	0.31 ± 0.03
Na (mmol/L)	103.32 ± 6.71^**^	143.46 ± 4.35
K (mmol/L)	4.37 ± 1.23	3.46 ± 0.74
Cl (mmol/L)	68.15 ± 17.82^**^	111.06 ± 5.47
Ca (mmol/L)	2.71 ± 0.66	2.29 ± 0.19
P (mmol/L)	2.98 ± 0.73^**^	1.50 ± 0.22
Mg (mmol/L)	1.19 ± 0.10	1.23 ± 0.05
Blood urea nitrogen (mmol/L)	1.19 ± 0.23	0.99 ± 0.00
Creatinine (μmol/L)	30.00 ± 11.28^**^	13.71 ± 1.92
Ammonia (μmol/L)	592.24 ± 177.84^**^	231.60 ± 65.37
Glucose (mmol/L)	1.56 ± 1.54	2.53 ± 0.74
Triglycerides (mmol/L)	1.11 ± 0.51	1.08 ± 0.18
Lactate (mmol/L)	5.76 ± 4.12^**^	11.01 ± 2.58
pH	7.43 ± 0.25^**^	7.13 ± 0.05
pCO_2_ (kPa)	3.58 ± 0.90^**^	5.78 ± 0.82
tCO_2_ (mmol/L)	20.34 ± 8.17	15.66 ± 1.58
HCO_3_ (mmol/L)	19.42 ± 8.15	14.28 ± 1.31
Base Excess (mmol/L)	−4.84 ± 11.96^*^	−15 ± 1.65
Anion Gap (mmol/L)	15.25 ± 2.62^*^	8.66 ± 3.35
Albumin (g/L)	15.18 ± 1.98^**^	7.68 ± 2.39
Total protein (g/L)	40.89 ± 10.99^*^	31.54 ± 4.08
Alkaline phosphatase (μkat/L)	1.07 ± 0.43^**^	0.30 ± 0.18
Alanine aminotransferase (μkat/L)	0.92 ± 0.73^**^	0.10 ± 0.05
Aspartate aminotransferase (μkat/L)	3.89 ± 1.85^**^	1.30 ± 0.91
Lactate dehydrogenase (μkat/L)	10.41 ± 6.21	10.54 ± 2.27

*Values represent mean ± standard deviation*,

* *p< 0.05*,

** *p< 0.01 when comparing CEV-infected fish against the healthy control. While non-parametric testing was performed for analysis of anion gap, alkaline phosphatase, aspartate aminotransferase and lactate dehydrogenase, parametric tests were used in the rest of variables*.

**Figure 3:**
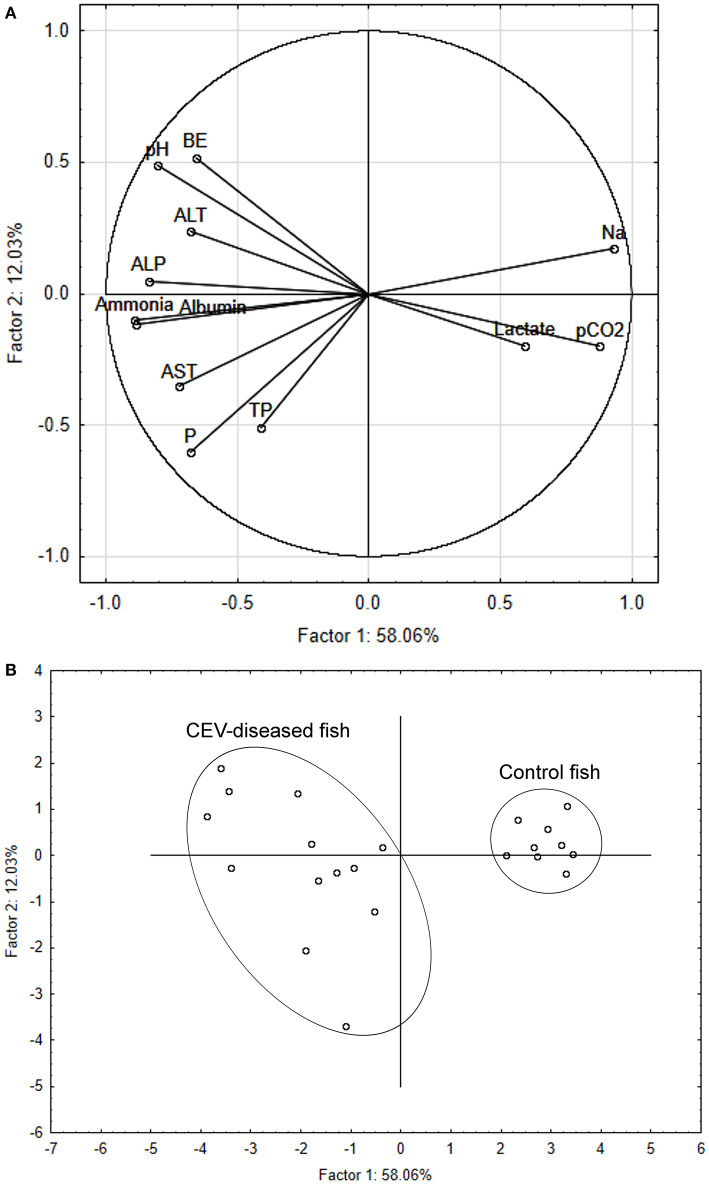
Principal component analysis (PCA) discriminating between carp edema virus (CEV)-infected and control fish (*Cyprinus carpio*). Component weight **(A)** and component score **(B)** plots are based on multiple blood chemistry variables. ALP, alkaline phosphatase; ALT, alanine aminotransferase; AST, aspartate aminotransferase; BE, base excess; Na, sodium; P, phosphorus; pCO_2_, partial dissolved carbon dioxide; TP, total protein.

The multivariate response pattern for blood chemistry variables showed a clear discrimination between CEV-infected and control carp, with variable component weights revealing separation between the two groups driven mainly by sodium, pH, partial dissolved carbon dioxide, ammonia and albumin (Figure 3A). Specimen samples plotted onto a two-dimensional space according to principal components 1 and 2 (Figure 3B) separated infected and healthy carp along the axis of the first principal component, which explained 58.06% of variation. In comparison, the second principal component only explained 12.03% of variation.

## Discussion

In the present study, we provide the first insight into the interaction between carp hosts and their pathogenic poxvirus under conditions of a natural CEV infection outbreak. We show that CEV infection resulted in severe metabolic disturbance, with significant differences from control healthy fish revealed by both univariate and multivariate analyses. The blood profile of infected fish was characterized by increased red blood cell associated hematology variables, electrolyte and acid-base imbalance, hypotonic dehydration, endogenous hyperammonaemia, and was also indicative of alterations in liver and kidney function. Importantly, many blood chemistry parameters showed major disruption, though some only differed against controls and were otherwise within normal ranges for common carp (14, 17).

Mortality amounted to 100% within <4 months of fish translocation, documenting high severity of the CEV infection outbreak and virulence of the agent. Likewise, quantification of viral loads confirmed high infection intensity of gills in fish showing clinical and pathological signs of disease. While the copy numbers of CEV-specific DNA in the branchial tissue were wide ranging, they corresponded with published data (1, 18). Adamek et al. (1) mentioned some discrepancy between clinical signs and gill pathology based on experimental infections. Contrary to this, the present study suggests that the viral load may be a suitable proxy for estimation of health deterioration in CEV-diseased carps. There is no quarantine pond at the farm under study. However, placing translocated carps to quarantine would not prevent the outbreak because it was most probably the stress of translocation plus the change in water temperature that promoted the outbreak among fish already carrying the agent (5).

Mechanisms controlling fluid and acid-base balance, electrolyte concentration, glucose level, and disposal of metabolic waste products are of vital importance for ensuring stable biochemical reactions and cellular processes (19). Thus, any loss in a fish's ability for homeostatic maintenance of such key physiological parameters will be damaging. The gills of fishes combine respiratory and excretory functions with osmotic, ionic, and acid–base regulation (20–23). While the gills and intestines are the principal organs for osmoregulation in fishes, the kidneys also contribute to fluid volume and electrolyte concentration, though to a lesser extent. Active uptake of ions across the intestines and gills is necessary to maintain hyperosmotic ionic homeostasis in all freshwater bony fishes (24), while chloride cells in the gills, rich in mitochondria, regulate the ionic and acid-base balance through exchange of Na^+^/H^+^/${\text{NH}}_{4}^{+}$ and Cl^−^/${\text{HCO}}_{3}^{-}$. Regarding the blood electrolyte concentrations measured in our CEV-infected carp, severe hyponatremia and hypochloraemia indicate gill function disruption, while hyperphosphatemia suggests alterations in kidney function (24). In general, manifestations of hyponatremia and hypochloraemia will include lethargy, fatigue, loss of appetite, muscle weakness, decreased consciousness, and/or coma as a sequel to cerebral edema. A decreased ability of fish to control extracellular ions fits well with the observed pathology of gills (the target organ of CEV replication), which included gill swelling, hyperplasia, clubbing of lamellae and necrosis (1), as well as the corresponding clinical signs (4, 5). The observed skin lesions may also contribute to a loss of electrolytes from the body (24). Enophthalmos, a pathological sign observed in CEV-infected fish (4, 5), can be attributed to hypotonic dehydration associated with a severe loss of electrolytes (Na^+^ and Cl^−^) from the body, combined with increased hematocrit, hemoglobin, red blood cell count, hyperproteinaemia, and hyperalbuminaemia (24). Given the reported absence of albumin in the common carp (25), the finding of hyperalbuminaemia may seem to be controversial and should be further pursued. Interestingly, elevation of high density lipoproteins might have been detected instead of carp albumin (25). On the other hand, serum chemistry reference ranges of albumin in *Cyprinus carpio* were published (14, 17, 26, 27).

We observed considerable alterations in the blood acid-base balance in CEV-infected fish (24). Contrary to our prediction of respiratory acidosis, higher pH and decreased pCO_2_ levels are indicative of respiratory alkalosis, probably due to hyperventilation and/or suppressed metabolism and cellular respiration. Hyperammonaemia, as a combined metabolic component of the acid-base balance, is probably responsible for the increase in base excess value observed in CEV-infected carp. Elevation of the anion gap, a measure of electrical neutrality, corresponds well with electrolyte imbalance, as well as marked alterations in albumin and lactate (24).

Ammonia (NH_3_), the major catabolite of proteins in freshwater fishes (21, 28), is dissolved in body fluids and the milieu of blood and/or is ionized to ammonium ions (${\text{NH}}_{4}^{+}$). In addition to temperature, the ratio of NH_3_/${\text{NH}}_{4}^{+}$ will depend on pH, with a higher pH favoring higher NH_3_ concentrations and increased toxicity due to the ease with which it can cross biological membranes. In line with this, the hyperammonaemia combined with alkalosis documented in our CEV-infected carp is likely to exert increased adverse effects due to the higher proportion of toxic unionized ammonia circulating in the blood. As reviewed in Ip and Chew (21), ammonia toxicity affects many cellular processes. It is known for its stimulatory effects on glycolysis through activation of phosphofructokinase I in cytosolic fluid, for interference with energy metabolism related to impairment of the tricarboxylic acid cycle in mitochondria and for disruption of ionic balance at both the cellular and organism levels. Furthermore, ionized ammonium ions can replace potassium ions (K^+^) in ion transporters and disrupt central nervous system electrochemical gradients. Indeed, passage of ammonia across the blood-brain barrier is why the brain is the principal target of ammonia toxicity in fish. According to Randall and Tsui (29), fish may be threatened by both environmental exposure and endogenous ammonia. In this study, low blood ammonia levels in healthy control fish from the same storage pond excluded the possibility of environmental ammonia contamination. Endogenous sources of ammonia in fish are associated with protein metabolism in the liver (amounting to 50–70% of total production), intestinal epithelial cells, kidneys, and skeletal muscles (28). Factors responsible for increased ammonia production may include postprandial metabolism, burst, and/or exhaustive exercise, starvation and stress (28, 30). Considering the lethargic behavior of our CEV-infected carp, which were fasted over the autumn period, starvation- and stress-induced catabolism, combined with the compromised excretory function of gills, would appear to be implicated in the elevated ammonia levels observed in this study. Moreover, ammonia toxicity can further deteriorate the condition of gills as both CEV and ammonia autointoxication result in gill necrosis (4, 5, 9, 28). Ammonia accumulation in fish blood, muscles, and brain, along with subsequent neuromotor dysfunction, are known to manifest as impaired locomotor performance (30), which corresponds with the clinical signs of lethargy and inactivity observed in CEV-infected carp (4, 5). Importantly, ammonia can also be considered a respiratory gas produced in ammoniotelic fishes at a rate equalling 10 to 20% of CO_2_ production or O_2_ uptake (22), stimulating ventilation by an increase in the ventilatory stroke volume, rather than the rate, in order to allow for ammonia excretion through the branchial surface (31).

Levels of blood urea nitrogen, another catabolite excreted through fish gills and an ammonia detoxification product, were comparably low in both CEV-infected and healthy control carp, again suggesting starvation. On the other hand, elevated levels of creatinine, which is mainly excreted through the kidney, suggests alterations in renal functioning in CEV-infected carp. Elevated levels for the enzymes ALP, ALT, and AST supports the finding of systemic responses seen in multiple organs, including the hepatopancreas, spleen, heart, and kidney, in addition to the gills (5).

Considering energy metabolism, no stress response was observable as there was only a non-significant reduction observed in glucose levels (32, 33). On the other hand, the triglyceride levels observed suggest no alteration to the lipid metabolism. Glycolysis, a key metabolic pathway supplying organisms with energy, may function both aerobically and anaerobically. In the absence of oxygen, it stops at pyruvate, which is reduced to lactate by lactate dehydrogenase (34). Elevation of lactate in blood indicates that the oxygen supply to tissues (e.g., white muscles in fish) is lower than that required for aerobic glycolysis (32). However, the decreased lactate levels observed in our study indicate that CEV-infected fish were not exposed to hypoxic stress (33), suggesting that, aside from gill pathology, oxygen demand was significantly lowered due to the carp's lethargic inactivity and disrupted hypometabolic condition. Unfortunately, blood oxygen saturation was not measured in the present study. In contrast to our findings indicating an absence of hypoxic stress, Lewisch et al. (5) reported clinical signs of dyspnoea and hypoxia in association with CEV, meaning that carp clustered at the inflow and utilized air-breathing at the water's surface.

While infectious agents are known to elicit disruption of blood homeostasis, multiple physical, chemical and biological stressors may often be combined (35–39), making it difficult to disentangle the link between infection-associated pathophysiological mechanisms of morbidity and mortality (40, 41). Interestingly, loss of osmoregulatory functioning in the gills, gut and kidneys are thought to contribute to mortality in carp affected with another viral agent, the koi herpes virus (34), which none of our samples was positive for. However, blood chemistry data for carp infected with koi herpes virus are limited (42), preventing detailed comparison.

To conclude, our blood profile study increases our understanding of CEV disease in carp, explaining how impaired respiratory and excretory functioning of gills, together with osmotic, ionic and acid-base disruption, exert complex adverse effects on the ability of fish to maintain homeostasis and support essential bodily functions. Further research should aim to quantify levels of infection intensity, the extent of gill damage and neurological abnormalities associated with neurotoxicity of ammonia as well as factors of fish susceptibility and conditions promoting disease manifestation.

## Data Availability Statement

The original contributions generated for the study are included in the article/Supplementary Material, further inquiries can be directed to the corresponding author/s.

## Ethics Statement

The animal study was reviewed and approved by The Ministry of Agriculture of the Czech Republic (permission No. MZe 1842). Written informed consent was obtained from the owners for the participation of their animals in this study.

## Author Contributions

JP and MP conceived the study and analyzed the data and wrote the first draft of the manuscript. JM and MP acquired funding. LP, IP, HM, IM, HB, JB, and MB performed field and/or laboratory investigations. All authors contributed critical comments on manuscript drafts and gave final approval for its publication.

## Conflict of Interest

The authors declare that the research was conducted in the absence of any commercial or financial relationships that could be construed as a potential conflict of interest.
