# Supplementary material for: Carp Edema Virus Infection Is Associated With Severe Metabolic Disturbance in Fish
Source: Front Vet Sci. 2021 May 19;8:679970. doi: 10.3389/fvets.2021.679970 (PMC8169968; doi:10.3389/fvets.2021.679970)
Supplement: Supplementary file 1 [file Data_Sheet_1.PDF]

fish group	Na (mmol/L)	K (mmol/L)	Cl (mmol/L)	tCO_2 (mmol/L)	urea (mmol/L)
CEV 1	99,0	5,10	53,60	20,00	
CEV 2	99,0	6,60	*49,90*	28,00	
CEV 3	100,2	5,20	51,20	13,00	
CEV 4	101,0	5,10	55,50	17,00	
CEV 5	99,0			33,00	
CEV 6	99,0			27,00	
CEV 7	101,0	4,40	73,00	14,00	1,10
CEV 8	106,0	3,70	79,00	19,00	1,60
CEV 9	110,0	2,80	92,00	6,00	1,20
CEV 10	99,0			14,50	
CEV 11	121,0	3,10	91,00	21,00	1,10
CEV 12	110,0	3,40		18,00	0,99
CEV 13	99,0			34,00	
Control 1	144,4	3,20	112,60	16,00	0,99
Control 2	144,8	4,20	112,00	18,00	0,99
Control 3	146,3	4,00	107,30	17,00	0,99
Control 4	140,1	2,60	109,60	15,00	0,99
Control 5	144,0	3,30	113,50	15,00	0,99
Control 6	135,4	3,30	106,20	17,00	0,99
Control 7	146,2	3,30	115,90	14,00	0,99
Control 8	150,1	2,50	120,40	13,00	0,99
Control 9	139,9	4,80	102,10	16,00	0,99

glucose (mmol/L)	haematocrit (L/L)	pH	pCO_2 (kPa)	HCO_3 (mmol/L)	base excess (mmol/L)
4,11	0,35	7,407	4,00	18,90	-6
1,38	0,35	7,487	4,73	26,90	3
0,47	0,36	7,146	4,71	12,20	-17
0,27	0,36	7,312	4,21	16,00	-10
1,22		7,881	2,31	32,50	15
1,52		7,704	2,80	26,20	6
4,78	0,34	7,418	2,71	13,10	-11
0,68	0,56	7,435	3,58	18,00	-6
0,54	0,34	6,993	2,98	5,40	-26
0,47		7,255	4,10	13,50	-14
0,63	0,20	7,336	4,93	19,10	-7
3,55	0,34	7,551	2,69	17,70	-5
0,65		7,791	2,89	33,00	15
3,10	0,25	7,117	5,94	14,40	-15
1,64	0,22	7,123	6,64	16,30	-13
3,16	0,22	7,062	7,13	15,20	-15
1,84	0,25	7,176	5,09	14,10	-14
3,73	0,35	7,051	6,48	13,50	-17
2,22	0,20	7,175	5,55	15,40	-13
2,75	0,26	7,115	5,27	12,70	-17
2,75	0,25	7,159	4,57	12,20	-17
1,63	0,16	7,167	5,43	14,80	-14

anion gap (mmol/L)	haemoglobin (g/L)	red blood cell count (T/L)	albumin (g/L)
	95,86	1,76	12,34
	96,40	1,85	12,45
	96,40	1,48	14,30
	97,31	1,78	18,43
	87,93	1,23	17,51
	80,30	0,92	17,38
19	96,65	2,20	13,35
13	113,29	2,47	15,27
15	101,26	2,04	13,60
	91,73	1,10	15,01
14	88,83	1,25	14,67
	96,65	2,30	17,00
	87,73	1,35	16,08
12	78,61	1,14	10,78
12	69,17	1,03	8,99
12	77,34	0,80	7,32
6	64,09	1,30	7,71
6	104,57	1,56	7,05
4	54,10	1,04	3,74
10	77,52	1,33	11,27
5	75,52	1,43	6,87
11	52,65	0,57	5,42

alkaline phosphatase (µkat/L)	alanine aminotransferase (µkat/L)
0,45	0,10
0,32	0,87
0,79	0,23
0,89	0,29
1,00	1,30
2,06	0,47
1,26	2,33
1,17	0,56
1,03	0,58
1,38	1,03
1,23	0,71
1,10	2,46
1,28	1,03
0,26	0,17
0,33	0,05
0,23	0,13
0,75	0,10
0,31	0,06
0,08	0,03
0,30	0,13
0,27	0,13
0,24	0,16

aspartate aminotransferase (µkat/L)	ammonia (µmol/L)	total protein (g/L)	P (mmol/L)
2,23	388,61	43,13	2,13
5,03	490,65	51,16	2,53
4,40	688,23	55,24	4,96
5,17	496,95	64,07	2,91
2,81	567,99	40,59	2,86
4,21	904,29	39,23	2,55
8,62	932,55	28,06	2,97
3,22	559,89	28,34	3,38
3,33	451,19	28,42	3,51
3,86	352,83	34,34	3,39
1,78	566,51	43,25	2,98
1,43	578,40	32,96	2,43
4,61	721,07	42,91	2,26
1,37	202,99	34,71	1,38
0,67	188,31	32,32	1,50
2,00	334,17	32,75	1,68
0,68	227,28	32,61	1,23
1,14	195,71	27,44	1,71
0,61	205,18	23,30	1,31
0,93	252,32	37,27	1,30
0,87	143,25	30,69	1,59
3,45	335,24	32,79	1,87

creatinine (µmol/L)	lactate dehydrogenase (µkat/L)	lactate (mmol/L)	Mg (mmol/L)
18,70	3,46	3,65	1,09
27,46	19,88	7,89	1,13
45,65	17,23	15,74	1,31
28,21	16,55	5,56	1,25
	17,81	2,71	
	5,14	4,44	
	16,96	7,62	
	5,94	2,39	
	6,15	2,30	
	5,73	0,24	
	3,99	4,71	
	9,89	10,73	
	6,65	7,04	
13,76	2,01	9,58	1,28
13,81	2,15	12,75	1,12
12,85	10,45	15,03	1,19
13,45	1,09	8,61	1,24
16,97	3,37	12,46	1,26
16,52	2,53	9,89	1,23
11,86	1,56	13,68	1,31
11,18	1,13	9,93	1,22
13,01	70,61	7,16	1,22

**triglycerides (mmol/L)**	**Ca (mmol/L)**
0,84	2,36
1,14	2,40
1,18	2,91
1,57	2,83
1,80	4,30
1,10	3,79
1,51	2,62
0,89	2,41
0,59	2,49
0,06	1,74
0,57	2,49
1,47	2,42
1,74	2,52
1,02	2,18
0,98	2,20
1,02	2,36
1,01	2,36
1,45	2,52
0,78	2,12
1,23	2,45
1,16	2,50
1,10	1,93
